# Hyperpolarisation of Mitochondrial Membranes Is a Critical Component of the Antifungal Mechanism of the Plant Defensin, Ppdef1

**DOI:** 10.3390/jof10010054

**Published:** 2024-01-07

**Authors:** Kathy Parisi, James A. McKenna, Rohan Lowe, Karen S. Harris, Thomas Shafee, Rosemary Guarino, Eunice Lee, Nicole L. van der Weerden, Mark R. Bleackley, Marilyn A. Anderson

**Affiliations:** 1 La Trobe Institute for Molecular Science, La Trobe University, Melbourne 3086, Australia; 2 Hexima Ltd., Preston 3072, Australia

**Keywords:** plant defensin, *Trichophyton rubrum*, *S. cerevisiae*, antifungal, Ppdef1, hyperpolarisation

## Abstract

Plant defensins are a large family of small cationic proteins with diverse functions and mechanisms of action, most of which assert antifungal activity against a broad spectrum of fungi. The partial mechanism of action has been resolved for a small number of members of plant defensins, and studies have revealed that many act by more than one mechanism. The plant defensin Ppdef1 has a unique sequence and long loop 5 with fungicidal activity against a range of human fungal pathogens, but little is known about its mechanism of action. We screened the *S. cerevisiae* non-essential gene deletion library and identified the involvement of the mitochondria in the mechanism of action of Ppdef1. Further analysis revealed that the hyperpolarisation of the mitochondrial membrane potential (MMP) activates ROS production, vacuolar fusion and cell death and is an important step in the mechanism of action of Ppdef1, and it is likely that a similar mechanism acts in *Trichophyton rubrum*.

## 1. Introduction

Defensins are defence proteins produced by all plants that are often encoded by multigene families with up to 200 members in some plant species [1,2,3,4,5]. They are small disulphide-rich cationic proteins of 45–54 amino acids that have a highly conserved structure despite the great variability observed in their sequences in terms of the eight conserved cysteine residues [6]. This sequence variability accounts for the wide range of functions and mechanisms of action exhibited by different plant defensins. They include antimicrobial activity against Gram-negative and Gram-positive bacteria, parasites, viruses and fungi, protein synthesis inhibition through interactions with nucleic acids, trypsin and α-amylase inhibition which interfere with insect digestion, roles in heavy metal tolerance, plant development and sexual reproduction and the inhibition of ion channels in mammalian and plant cells [6,7,8]. Plant defensins are most commonly described as antifungal molecules, and even here, the sequence hypervariability contributes to at least six different mechanisms of action [8]. Defensins have seven loops, defined as the sequences between the four conserved disulphide bonds, which have a major role in the biological activity of defensins [6,8]. Loop 5 is the flexible region between β-strands 2 and 3 that is essential for the specificity and function of plant defensins. It is exposed on the surface of a protein, is highly variable in sequence and has a major role in antifungal activity and lipid-binding activity [6,8,9,10,11,12,13,14].

The mechanism of action of a small number of plant defensins has been elucidated, although these mechanisms are complex and only partially understood [8,14]. Plant defensins have multiple targets and not all of these have been identified [8]. The initial step in the antifungal mode of action is the interaction with the fungal cell surface. Here, defensins can be divided into two groups. The first group acts extracellularly by binding to the cell wall components, membrane receptors and/or to specific membrane lipids such as sphingolipids or phospholipids. An example is RsAFP2 which binds to the sphingolipid glucosylceramide in the fungal cell wall, inducing cell wall stress and activating the MAPK cell wall integrity pathway [15]. The second group also binds to cell surface receptors and membrane lipids and includes NaD1, SBI6, NbD6, MtDef4 and PsD1. They differ from the first group in their ability to traverse the cell wall and plasma membrane and enter the fungal cell where they act on cytoplasmic targets [16,17,18,19,20,21]. Loop 5 is often responsible for the antifungal activity of most plant defensins and specific binding to certain lipids. For example, defensins MtDef4, NaD2, ZmD32 and NbD6 have identical loop 5 sequences, RGFRRR, and all bind to the lipid phosphatidic acid (PA) [11,20,22]. In contrast, NaD1 has the loop 5 sequence SKILRR which binds preferentially to phosphatidylinositol 4,5 bisphosphate (PI(4,5)P_2_) in addition to PA [9,23].

Here, we describe the mechanism of action for the defensin Ppdef1 from the plant *Picramnia pentandra*, otherwise known as Florida bitter bush. The sequence of this plant defensin was identified from genomic databases and was selected based on its unique sequence. A search of the NCBI non-redundant database of approximately 1000 plant defensin sequences revealed that the sequence of Ppdef1 (KVCTKPSKFFKGLCGTDGA CTTACKEGLHSGYCQLKGFLNSVCVCRKHC) is very different to that of other defensins with only 48% similarity with the closest member and 35% sequence similarity to characterized defensins. Furthermore, this reduced to 16% when the highly conserved cysteines and glycine residues essential to the defensin fold were removed. The structural analysis of Ppdef1 revealed an extended loop 5 that is unlike any other plant defensin with a characterized mechanism of action as well as the other plant defensins in the database [24]. Like most plant defensins, Ppdef1 has excellent antifungal activity against a suite of human fungal pathogens including dermatophytes that infect skin and nails [24]. Despite its size (~5 kDa), Ppdef1 can penetrate human nails more efficiently than other smaller antifungal drugs and rapidly kills the fungal pathogen *Trichophyton rubrum* when growing on nails or in the presence of keratin [24]. Ppdef1 is also known as pezadeftide (INN designation) and HXP124 and has been used topically in Phase 1 and 2 clinical trials for the treatment of onychomycosis with some efficacy (ACTRN12618000131257 and ACTRN12620000697987). The Ppdef1 name is used in this manuscript in accordance with the defensin-naming convention (plant initials defensin 1) [25,26,27].

Here, we have used *Saccharomyces cerevisiae* as a model system to demonstrate that Ppdef1 rapidly enters and kills fungal cells. We then used the genetic and biochemical tools that are available for use in *S. cerevisiae* to identify potential targets within fungal cells and to monitor the changes in cell morphology prior to cell death. The screen identified the involvement of the mitochondria in the mechanism of action of Ppdef1, and further experiments revealed that Ppdef1 causes the hyperpolarisation of the mitochondrial membrane potential (MMP). Discoveries regarding the mechanism of Ppdef1 that were made using *S. cerevisiae* were used to design experiments to show that many of the molecular events take place when the major causative agent of onychomycosis, *T. rubrum,* is exposed to Ppdef1.

## 2. Materials and Methods

### 2.1. Fungal and Bacterial Strains Used and Culture Conditions

The wild-type *S. cerevisiae* strain BY4741 (MATa his3D1leu2D0 met15D0 ura3D0) and the non-essential gene deletion library were purchased from Thermo Fisher Scientific (Victoria, Australia). All of the non-essential gene deletion mutants were replicated in YPD (1% yeast extract, 2% peptone, 2% dextrose) supplemented with G418 and stored in 25% glycerol 96-well microtiter plates at −80 °C. *S. cerevisiae* was maintained on YPD agar at 30 °C, and overnight cultures were grown in YPD. The *Trichophyton rubrum* (isolate 13-34934613) and *Trichophyton mentagrophytes* (isolate 14-35867448) fungi were supplied by the National Mycology Reference centre (NMRC, Adelaide, Australia) and were maintained on V8 agar at 25 °C. *Candida troplicalis* (ATCC 750), *Candida glabrata* (ATCC 90030), *Candida auris* (17-470121) and *Candida krusei* were sourced from the NMRC (Australia). *Candida albicans* (ATCC MYA-2876) was a gift from Dr Ana Travern (Monash University, Victoria, Australia). All *Candida* isolates were maintained on YPD agar at 30 °C. *Microsporum fulvum* (05-202551) was maintained on ½ strength Sabouraud dextrose agar (½ SDA), and spores were grown in ½ strength potato dextrose broth (½ PDB) at 25 °C. *Fusarium graminearum* (PH-1) and *Fusarium oxysporum* f.sp*. vasinfectum* (isolate 5176) were maintained on synthetic nutrient poor, SNP, agar (0.1% KH_2_PO_4_, 0.1% KNO_3_, 0.1% MgSO_4_·7H_2_O, 0.05% KCl, 0.02% glucose, 0.02% sucrose, 2% agar) at 25 °C, and spores were cultured in ½ PDB at 25 °C. *Crytptococcus neoformans* (KN99), *Cryptococcus gattii* R265) and *Aspergillus niger* (5181) were supplied by Dee Carter (University of Sydney, Sydney, Australia). The *Cryptococcus* sp. was maintained on YPD agar, and overnight cultures were grown in YPD medium at 30 °C. For the *Aspergillus* strain, spores were added to ½ PDB and allowed to grow at room temperature for 14 days. The bacterial strains *Escherichia coli* TOP10, *Staphylococcus aureus* ATCC 9144, *Pseudomonas aeruginosa* PAO1 and *Bacillus subtilis* (La Trobe University isolate, Victoria, Australia) were all maintained on LB agar (1% tryptone, 0.5% yeast extract, 1% NaCl, 1.5% agar) plates, and overnight cultures were grown in LB medium at 37 °C.

### 2.2. Recombinant Expression of Plant Defensins Using Pichia Pastoris

Ppdef1 was produced using the methylotrophic yeast *Pichia pastoris* and purified as described by van der Weerden et al. [24]. NaD1 and NaD2 were isolated from the flowers of *Nicotiana alata* using the methodology described in Lay et al. [28]. NbD6 and ZmD32 were expressed and purified using the pPIC9 and pPINK expression systems in *P. pastoris*, respectively, as described in Lay et al. and Hayes et al. [23,29]. An alanine was added to the N-terminus of Ppdef1, NbD6 and ZmD32 sequences to enhance cleavage by Kex2 during expression in yeast. The mass and purity of the purified proteins were confirmed using SDS-PAGE, MALDI-TOF-MS and RP-HPLC.

### 2.3. Fungal and Bacterial Growth Inhibition Assays

Antifungal activity of Ppdef1 was assessed against an array of fungal pathogens including *Trichophyton* sp., *Cryptococcus* sp., *Aspergillus* sp., *Candida* sp., *Fusarium* sp., *M. fulvum* and *S. cerevisiae* (BY4741) using the microbroth dilution assay as described in Hayes et al. [29]. Defensins were assessed in a twofold dilution series from 50 µg/mL. IC50 µg/mL was defined as the concentration which inhibited 50% growth. Bacterial pathogens *E. coli*, *B. subtilis*, *P. aeruginosa* and *S. aureus* were treated with Ppdef1 in a microplate assay as described in Kerenga et al. [22]. Three independent replicates were used for each assay.

### 2.4. Alignment of Defensin Sequences

The sequences of 996 plant defensins including those with partially known mechanisms of action were collected from the non-redundant database and aligned using the Cysbar webserver [30]. The percentage conservation was calculated for each residue. The sequences of plant defensins with partially known mechanisms of action, including the sequence of CDL12_12515 which has an unknown mechanism of action but 48% similarity with Ppdef1 identified through a BLASTp search were used to construct a CLUSTALW alignment using the program MUSCLE-Multiple sequence alignment. Sequence identity and the conservation of residues between proteins was also calculated using MUSCLE (https://www.ebi.ac.uk/Tools/msa/clustalo/, accessed in 1 September 2023).

### 2.5. Beta-Glucan and Chitin-Binding Assays

Polysaccharide-binding assays were performed as described by Bleackley et al. [31]. All experiments were performed in triplicate. In brief, the test defensins were added to increasing amounts of insoluble polysaccharide yeast β-glucan (Megazyme, Sydney, Australia) or chitin from shrimp cells (Sigma Aldrich, St. Louis, MI, USA), and the mixture was incubated on a rotator wheel at room temperature for 30 min before centrifugation at 17,000× *g* to pellet the insoluble polysaccharide. The supernatant was then removed, and 25 µL of this supernatant was analysed using SDS-PAGE. The test defensins (unbound) were visualised with RAPIDstain (Calbiochem, Victoria, Australia) as per the manufacturer’s protocol and imaged with Gel Doc (BioRad, Hercules, CA, USA).

### 2.6. Lipid-Binding Assays

Lipid-binding assays were performed using PIP StripsTM or SphingoStripsTM (lipid–protein interaction assay, Echelon Biosciences, Salt Lake City, UT, USA) as described in [9,22]. Ppdef1’s lipid binding was detected using polyclonal antibodies generated in rabbits specific to the Ppdef1, DmAMP1 or NaD1 defensins using Gel Doc (Biorad). The PIP Strips are hydrophobic membranes with 100 pmol of the following lipids: Lysophosphatidic acid (LPA), Lysophosphocholine (LPC), Phosphatidylinositol (PI), Phosphatidylinositol (3)-phosphate (PI(3)P), Phosphatidylinositol (4)-phosphate (PI(4)P), Phosphatidylinositol (5)-phosphate (PI(5)P), Phosphatidylethanolamine (PE), Phosphatidylcholine (PC), Sphingosine 1-Phosphate (S1P), Phosphatidylinositol (3,4)-bisphosphate (PI(3,4)P_2_), Phosphatidylinositol (3,5)-bisphosphate (PI(3,5)P_2_), Phosphatidylinositol (4,5)-bisphosphate (PI(4,5)P_2_), Phosphatidylinositol (3,4,5)-trisphosphate (PI(3,4,5)P_3_), Phosphatidic acid (PA) and Phosphatidylserine (PS). SpingoStrips are hydrophobic membranes with 100 pmol of the following lipids: Sphingosine (S), Sphingosine 1-phosphate (S1P), Phytosphingosine (PhS), Ceramide (Cer), Sphingomyelin (SM), Sphingosylphosphorylcholine (SPC), Lysophosphatidic Acid (LPA), Myriosine (Myr), Monosialoganglioside-GM1 (GM1), Disialoganglioside-GD3 (GD3), Sulfatide, Psychosine, Cholesterol (C), Lysophosphatidylcholine (LPC) and Phosphatidylcholine (PC). A 5 µL volume of defensin (5 µg/mL) was spotted onto the membrane as a positive control and allowed to dry before blocking.

### 2.7. Confocal Microscopy 

#### 2.7.1. Site-Specific Labelling of Ppdef1 with 5FAM

The fluorescent tag 5FAM was linked to the C-terminus of Ppdef1 using an asparaginyl endopeptidase (AEP) from *Oldenlandia affinis*. Ppdef1 with the C-terminal AEP-recognition sequence TRNGLP was expressed in *P. pastoris* and purified as described in Lay et al. [23]. The asparaginyl endopeptidase *Oa*AEP3 was recombinantly expressed in *E. coli* and purified as described by Harris et al. [32]. The yield and purity of the expressed proteins was evaluated using MS and SDS PAGE followed by Western blotting and staining with Instant Blue (Abcam, Victoria, Australia). Protein concentrations were estimated with bicinchoninic acid assay as per the manufacturer’s instructions. GLK-5FAM peptide was ordered from GL Biochem (Shanghai, China) and reconstituted with sterile ultra-pure water (11.9 mM). The final concentrations of GLK-5FAM (280 µM) and Ppdef1-TRNGLK (140 µM) were incubated with r*Oa*AEP3 (0.066 µM) in 10× activity buffer (500 mM sodium acetate, 5 mM NaCl, 10 mM EDTA, pH 5.0) in a 4 mL reaction volume for 30 min at 25 °C. The enzyme was deactivated by heating to 70 °C for 10 min and with the addition of trifluoroacetic acid (TFA) to 0.1%. The filtered crude deactivated reaction mixture was loaded onto an analytical Agilent ZORBAX C8 reversed-phase column (5 µm, 4.6 × 150 mm) and separated on an Agilent analytical HPLC system with 0–40% Buffer B (60% acetonitrile, 0.89% TFA) for 0–5 min, 40–70% Buffer B for 5–25 min and 70–100% Buffer B for 25–27 min. Fractions containing the 5FAM-tagged Ppdef1 and unconjugated proteins were collected, analysed with microTOF Q (Bruker, Victora, Australia) and lyophilised as described by Harris et al. [32]. Ppdef1-TRNGLP was also labelled with GLK-TAMRA (GL Biochem) using the same procedure.

#### 2.7.2. Treatment of Vacuole-Stained Yeast Cells with FAM-Labelled Ppdef1

The vacuoles of *S. cerevisiae* cells were prestained with FM4-64 as described in Vida et al. [33]. The cells in CDAA medium (½ Czapek Dox, ½ drop out mix complete amino acids, United States Biological, pH 5.0, Salem, USA) were then loaded into the wells of a CellASIC microfluidic plate (Y04C, Merck, Victoria, Australia), prewashed with sterile ultra-pure water using the CellASIC ONIX microfluidic system (Merck, Victoria, Australia), and examined using a Zeiss LSM 780 laser scanning confocal microscope (Carl Zeiss AG, Jena, Germany). To confirm FM4-64 vacuolar staining, the pre-labelled cells were observed using an LDC apo 40× water immersion/1.1 Korr M27 objective with FM4-64 excitation at 561 nm and fluorescence detection at 591 nm. The cells were subsequently treated with 10 µM FAM-labelled defensin and monitored for 40 min with FAM excitation at 488 nm and fluorescence detection at 530 nm. Confocal fluorescence and corresponding bright-field images were collected simultaneously and analysed using the Zen 2.1 and Zen 2.0 lite software, respectively.

#### 2.7.3. Treatment of *T. rubrum* Hyphae with TAMRA-Labelled Ppdef1 for Visualisation of ROS Production or Permeabilization with SYTOX Green

*T. rubrum* spores were prepared from conidia grown on V8 agar at 28 °C for four weeks under fluorescent light. Conidia were collected by gentle scraping of the surface of the plate and flushing with sterile ultra-pure water before filtration through a sterile facial tissue. Collected conidia were quantified using a hemocytometer and diluted to 10^5^ spores/mL in ½ potato dextrose broth (PDB). Spores were loaded with ½ PDB into the barrier traps and prewashed with sterile ultra-pure water, in a CellASIC microfluidic plate (Y04T, Merck) using the CellASIC ONIX microfluidic system. The plates were sealed with a breathable membrane (Sigma Aldrich) and incubated at 28 °C overnight (ON) for hyphal growth. Hyphae were washed with CDAA medium and treated with Ppdef1-TAMRA. Hyphae were initially treated in row 1 with 10 µM Ppdef1 in the presence of SYTOX green (0.5 µM) and examined using a Zeiss LSM 780 laser scanning confocal microscope. Hyphae were observed using an LDC apo 40× water immersion/1.1 Korr M27 objective. TAMRA excitation was at 561 nm with fluorescence detection at 591 nm, and SYTOX was excited at 488 nm with fluorescence detection at 530 nm. Hyphae were monitored during treatment for up to 40 min. Confocal fluorescence and corresponding bright-field images were collected simultaneously and analysed using the Zen 2.1 and Zen 2.0 lite software, respectively. Hyphae in row 2 were subsequently treated with 10 µM Ppdef1-TAMRA in the presence of dihydrorhodamine (DHR 123, Thermo Fisher Scientific) for the detection of reactive oxygen species (ROS) and monitored with confocal microscopy as described for the SYTOX procedure above.

### 2.8. Membrane Permeabilization of Ppdef1

Membrane permeabilization was assessed by incubating *S. cerevisiae* cells with the non-permeable fluorescent dye SYTOX green, 0.5 µM (Thermo Fisher Scientific), before and after the addition of defensin (final peptide concentration: 0 µM, 10 µM, 20 µM or 30 µM) as described by van der Weerden et al. [34]. Fluorescence was measured every 2 min with a Spectramax M5e microplate reader (Molecular Devices, San Jose, CA, USA) with excitation and emission wavelengths of 488 nm and 538 nm, respectively.

### 2.9. Detection of Reactive Oxygen Species Using Flow Cytometry

The induction of ROS production was examined by incubating *S. cerevisiae* cells with dihydrorhodamine 123 (DHR 123; Thermo Fisher Scientific), before the addition of 10 µM of the defensin Ppdef1, followed by analysis using flow cytometry [29]. Briefly, *S. cerevisiae* cells were diluted from an overnight culture to OD600 of 0.1 in CDAA, and 80 µL of this diluted culture was aliquoted to wells of a round bottom microtitre plate with 25 µM DHR 123. Defensin was added (20 µL), and the cells were incubated for 1 h 30 °C kept stationary in the dark. The cells were analysed using the BD FACS II Canto cytometer (Becton Dickinson, Franklin Lakes, NJ, USA)as described by Hayes et al. [29], and the data was analysed with Weasel v3.0 (Walter and Eliza Hall Institute, Victoria, Australia).

### 2.10. Detection of Reactive Oxygen Species Using Flow Cytometry in the Presence of Ascorbic Acid

*S. cerevisiae* cells were monitored for ROS production in the presence of sodium ascorbate using flow cytometry as described by Hayes et al. [29]. Briefly, the BY4741 wild-type strain was grown on YPD agar and used to inoculate 4 mL of YPD before incubation with shaking (300 rpm, 30 °C, ON). The cells were diluted to OD600 0.1 with CDAA and added to wells of a round bottom microtitre plate containing 5 µM or 50 µM final concentration of ascorbate and a final concentration of 5 µM, 10 µM or 20 µM Ppdef1 in the presence of DHR123 in a 200 µL volume in triplicate. The cells were assessed using flow cytometry with excitation at 488 nm and emission detected with the 530/30 filter.

### 2.11. Screening of the S. cerevisiae Non-Essential Gene Deletion Set

The gene deletion collection was purchased from Thermo Fisher Scientific and stored at −80 °C. It consists of approximately 5000 yeast strains, each with a specific barcode incorporated during the deletion procedure [35]. These barcodes facilitate analysis of each strain in the entire deletion collection in a single pooled experiment. The gene deletion library was plated onto YPD agar supplemented with G418, and colonies were pooled as described by Smith et al. [36] and Parisi et al. [16]. Both the pooled deletion library and the wild-type (BY4741) cells were treated with Ppdef1 defensins as described by Parisi et al. [16]. Briefly, an ON culture of BY4741 was diluted to an OD600 of 0.4 with CDAA medium, and a 1 mL frozen aliquot of the pooled library with an OD600 of 50 was thawed and diluted to an OD600 of 0.4 with CDAA. Aliquots (5 mL) of the freshly diluted pooled library and the BY4741 cells were transferred to 4 culture tubes (Nunc), respectively. Ppdef1 was added at a concentration that inhibits 50% of the growth of the BY4741 cells. The treatments were performed in triplicate for the deletion pool and the BY4741 cells for 16 h at 30 °C with agitation (230 rpm). The untreated and defensin-treated pools were then harvested using centrifugation at 3220× *g* for 20 min, and genomic DNA was isolated. The genomic DNA was amplified to incorporate an Illumina adapter sequence followed by a unique index sequence for discrimination between the untreated and treated pools after next generation sequencing. The PCR amplicons were purified using gel extraction and quantified using a Qubit dsDNA HS (high sensitivity) assay kit (Thermo Fisher Scientific), according to the manufacturer’s protocol. Purified pooled DNA was prepared according to the manufacturer’s protocol (Illumina, San Diego, CA, USA) alongside the PhiX (Illumina) control (10%) and loaded into the MiSeq cartridge. The sequencing was performed on an Illumina MiSeq sequencer using v3 150-cycle paired-end chemistry. The relative abundance of each strain-specific barcode in a pool was counted and compared using next generation sequencing. Paired-end Illumina reads in FASTQ format were checked for quality issues, and read pairs were merged into a single sequence. Reads were aligned to the PhiX sequence and filtered (NC_001422). Remaining reads were demultiplexed into groups corresponding to the sample libraries. Trimmed barcodes were then matched to the barcode list, and raw barcode counts were exported. Statistical analysis of the barcode abundances was performed for each library to calculate normalized abundances for each treatment, and fitness scores were determined. The genes with a fitness score of >1.5 and >−1.5 for each treatment were analyzed using FunSpec (http://funspec.med.utoronto.ca/, accessed in 1 August 2023) [37]. The antifungal activity of Ppdef1 was assessed against ten unique strains identified from the screen with increased resistance to Ppdef1. Antifungal assays were performed with 1 µM Ppdef1 as described by Parisi et al. [16] using CDAA medium.

### 2.12. Detection of Mitochondrial Hyperpolarisation Using Flow Cytometry

Change in the mitochondrial membrane potential was monitored in *S. cerevisiae* cells before and after treatment with Ppdef1 or NaD1 using the red-orange, fluorescent dye Tetramethylrhodamine, methyl ester (TMRM, Thermo Fisher Scientific) that is sequestered by active mitochondria. BY4741 cells were grown from a glycerol stock at 30 °C ON on YPD agar, and used to inoculate 5 mL YPD broth, with shaking, at 300 rpm and at 30 °C ON. Cells were diluted to an OD600 of 0.1 in YPD and incubated with 25 µM TMRM at 30 °C for 1 h with gentle mixing every 15 min. Cells were centrifuged at 700× *g* for 3 min, and the YPD medium was replaced with 1 mL of CDAA supplemented with 25 µM TMRM. The cells were diluted to OD600 of 0.3 in CDAA supplemented with TMRM, and 180 µL aliquoted to wells of a round bottom microtitre plate containing 0 µM, 1.0 µM, 2.5 µM, 5.0 µM or 10 µM Ppdef1, NaD1 or 10 µM final concentrations of CCCP in a final volume of 200 µL in triplicate. The cells were assessed for fluorescence using flow cytometry with excitation at 488 nm, and the emission was detected with the 670H emission filter immediately before an incubation for 15 min at room temperature when the cells were assessed again.

## 3. Results

### 3.1. Broad Spectrum Antifungal Activity of Ppdef1

Ppdef1 has broad spectrum activity against several fungal pathogens. The activity of Ppdef1 was compared to the activity of the plant defensins NaD1, ZmD32, NbD6 and NaD2 against several human pathogenic fungi, two plant pathogens and four different bacterial strains (Table 1). Ppdef1 was as or more potent than NaD1, ZmD32, NbD6 or NaD2 against most of the fungal pathogens tested except for *C. glabrata*, *C. gattii* and the agricultural pathogen *F. graminearum*. Ppdef1 was the most active defensin against *C. auris* but had no activity against the bacterial pathogens when tested up to 200 µg/mL. In contrast, ZmD32 was active against all the bacterial pathogens tested with an IC50 of <10 µg/mL.

### 3.2. Sequence Analysis

**Figure 1:**
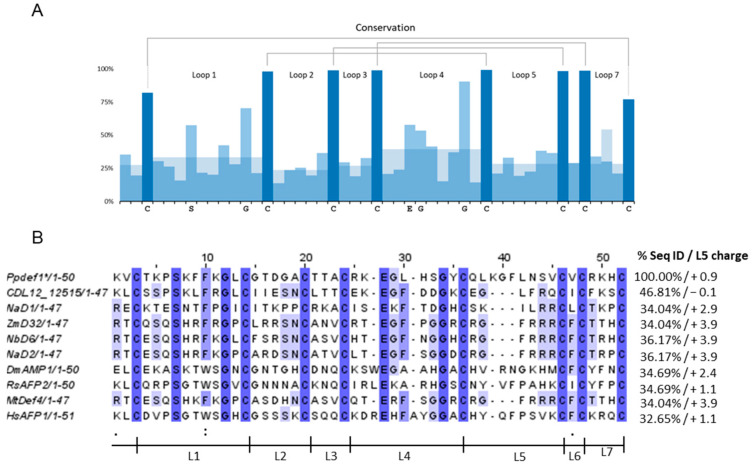
Ppdef1 shares little sequence similarity with other plant defensins. (**A**) Percent conservation of residues between the ~1000 plant defensin sequences. The black lines connecting the conserved cysteine residues represent the disulfide bonds. Cysteine residues are highlighted in deep blue. (**B**) MUSCLE alignment of native sequences for Ppdef1 to plant defensins with partially characterised mechanisms of action. Conserved residues are coloured deep purple, and similar amino acids are light purple. Gaps have been inserted to maximize alignment. Sequence identity and predicted charge of the loop 5 sequence at pH 7.0 are listed on the right. ‘.’ Indicates residues with weakly similar properties, and ‘:’ indicates residues with strongly similar properties. The black line beneath the alignment shows regions between cysteine residues defined as loops 1–7 (Ppdef1—*Picramnia pentandra*; NaD1—*Nicotiana alata* Q8GTM0; ZmD32—*Triticum aestivum* 6DMZ_A; NbD6—*Nicotiana benthamiana* [16]; NaD2—*Nicotiana alata* AOD75394; DmAMP1—*Dahlia merkii* AAB34972; RsAFP2—*Raphanus sativus* P30230; MtDef4—*Medicago truncatula* 2LR3_A; and HsAFP1—*Heuchera sanguinea* AAB34974.1).

The conservation of amino acid sequence across ~1000 plant defensins was analysed (https://ts404.shinyapps.io/defspace/, accessed in 1 August 2023) [19]. The percent conservation of the known conserved cysteines ranged from 77 to 100% across all defensins, and the conservation of the glycine residues was 70–90% (Figure 1A). The conservation of amino acids within each of the loops was in the range of 14–58% (Figure 1A). A sequence alignment of Ppdef1 with eight plant defensins with known mechanisms of action revealed that these defensins share 37% sequence identity (Figure 1B). Another plant defensin CDL12_12515 (*Handroanthus impetiginosus* PIN14865.1) was included in the alignment because it is the most closely related defensin in the database, but its mechanism of action is unknown. It has a very different loop 5 sequence to Ppdef1. Indeed, loops 2, 3, 4 and 5 of each of the defensins in the alignment share little or no sequence identity with Ppdef1. Furthermore, loop 5 on Ppdef1 is longer (9 aa) than loop 5 on most plant defensins (6–8 aa) (Figure 1B). The estimated charge for loop 5 sequences at pH 7.0 was low for Ppdef1 and CDL12_12515 at +0.9 and −0.1, respectively, and highest at +3.9 for ZmD32, NaD2, NbD6 and MtDef4, which have the loop 5 sequence RGFRRR.

### 3.3. Lipid-Binding Analysis

**Figure 2:**
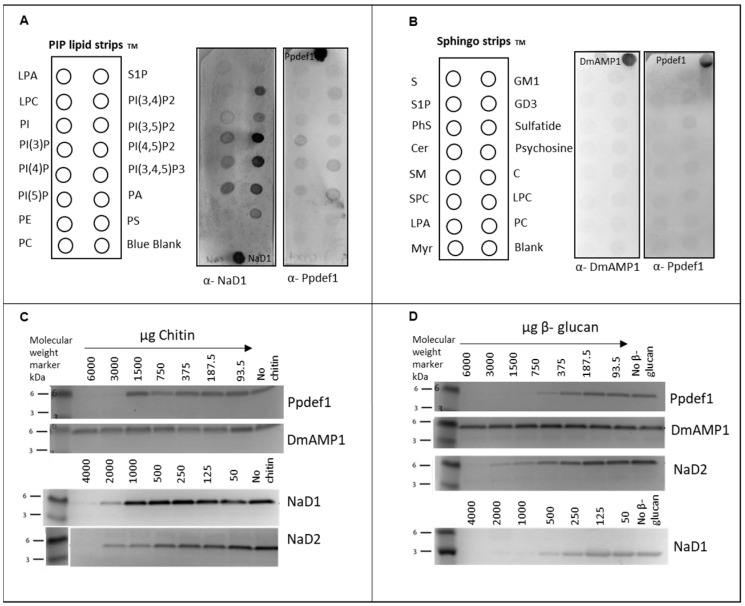
Lipid strip and carbohydrate binding. (**A**) PIP stripTM probed with NaD1 and Ppdef1. NaD1 bound to PI(3,4)P_2_, PI(4,5)P_2_, PI(3,4,5)P_3_, PA and PI(5)P, and Ppdef1 did not bind. NaD1 and Ppdef1 (0.025 μg) were used as positive controls. (**B**) SphingoStripTM probed with DmAMP1 and Ppdef1. DmAMP1 and Ppdef1 did not bind to any of the lipids on the SphingoStripTM. Purified defensins (0.025 μg) were used as positive controls. (**C**,**D**) Amount of defensin remaining in the supernatant after incubation with increasing amounts of insoluble polysaccharide. Defensins were detected using Western blot with specific antibodies. (**C**) NaD1, NaD2, DmAMP1 and Ppdef1 binding to chitin. (**D**) NaD1, Ppdef1, NaD2 and DmAMP1 binding to yeast β-glucan.

Since loop 5 is generally the sequence on plant defensins that is involved in specific lipid binding [9,20,23] and Ppdef1 has a very different loop 5 sequence, we used lipid strips to examine whether Ppdef1 binds to lipids. Ppdef1 had no affinity to lipids on either the PIP StripTM or the SphingoStripTM lipid–protein interaction assays (Figure 2A,B). In contrast, NaD1 which was used as a positive control bound preferentially to PI(4,5)P_2_ with some binding to other phosphatidylinositol monophosphates (Figure 2A). DmAMP1 did not bind to lipids on the SphingoStripTM as expected because DmAMP1 binds preferentially to M(IP)_2_C [38,39] which was not represented on the SphingoStripTM (Figure 2B). 

Some defensins bind to polysaccharides in fungal cell walls [10,31]; therefore, the binding of defensins to cell wall carbohydrates was also examined. Ppdef1, NaD1 and NaD2 were bound to high concentrations of chitin and yeast β-glucan, whereas DmAMP1 did not bind to either polysaccharide (Figure 2C,D).

### 3.4. Confocal Microscopy to Visualise Intracellular Ppdef1

**Figure 3:**
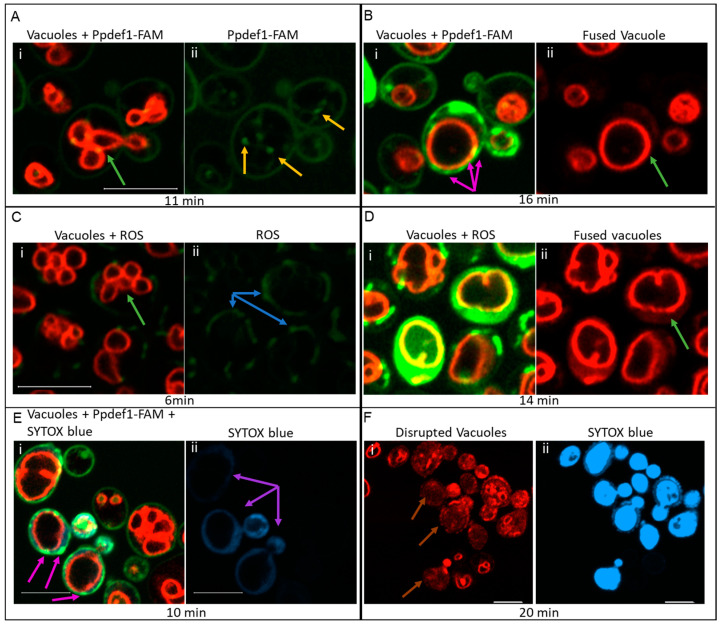
Uptake of Ppdef1-FAM into *S. cerevisiae* cells, effect on ROS production, vacuole morphology and cell permeability. *S. cerevisiae* cells, pre-labelled with FM-464 vacuolar stain (red), were treated with Ppdef1-FAM (green, (**A**,**B**,**E**,**F**)) or unlabelled Ppdef1 (**C**,**D**) in a CellASIC ONIX microfluidic plate. The labelled defensin (green), vacuolar morphology (red), ROS (green) and permeabilization (blue) were monitored over time using confocal microscopy. In one experiment, the uptake of Ppdef1-FAM was monitored (**A**,**B**). Ppdef1-FAM bound to the surface of the cell ((**Ai**) red and green merged channels) within 11 min and had moved into the cytoplasm ((**Aii**) green channel only) highlighted with yellow arrows. At ~16 min, the vacuoles had fused into one large vacuole ((**Bi**) red and green merged channels; (**Bii**) red channel only) from multiple smaller vacuoles ((**Ai**) red and green merged channels) highlighted with green arrows. As the vacuoles fused, Ppdef1 was concentrated into punctate structures at the edge of the cell ((**Bi**)) indicated with pink arrows. In a second experiment, the production of ROS was monitored. Cells with pre-labelled vacuoles (red) were treated with Ppdef1 in another chamber of the microfluidic plate in the presence of DHR123, a ROS stain (green). ROS was produced in the mitochondria of these cells after 6 min ((**Ci**) merged green and red channels; (**Cii**) green channel only), and ROS is highlighted with blue arrows. At 14 min, the vacuoles fused into one larger vacuole (green arrow) ((**Di**) green and red merged channels; (**Dii**) red channel only). In a third experiment, the permeabilization of the membrane with SYTOX blue was monitored. Cells with pre-labelled vacuoles were treated with Ppdef1-FAM in another chamber of the plate in the presence of SYTOX blue to monitor the permeabilization of the membrane. Permeabilization occurred in these cells at 10 min at the time of vacuole fusion, allowing the migration of SYTOX blue into the cell ((**Ei**) red, green and blue merged channels; (**Eii**) blue channel only). Sytox indicated with purple arrows, (**Eii**), puncta highlighted with pink arrows, (**Ei**). The vacuole was subsequently disrupted, highlighted with brown arrows ((**Fi**) red channel only), and the cells were filled with SYTOX blue, indicating cell death, 20 min ((**Fii**) blue channel only). Scale bars represent 5 µm.

To determine whether Ppdef1 acts extracellularly or intracellularly, confocal microscopy was used to trace the location of Ppdef1 on and in *S. cerevisiae* cells that had been treated with Ppdef1-FAM. The *O. affinis* asparaginyl endopeptidase (*Oa*AEP1) was used to ligate a single fluorescent FAM molecule (green) to Ppdef1 via the C-terminus. This allowed a clearer imaging of the fluorescent peptide than other conjugation methods. The vacuoles of *S. cerevisiae* cells were pre-labelled with the red fluorescent dye FM-464 before treatment with Ppdef1-FAM. Ppdef1 was detected at the cell surface approximately 4 min after addition to the cells, and continued to accumulate on the cell surface before it was detected throughout the cytoplasm at 11 min (Figure 3A).

Several defensins have been reported to induce the production of reactive oxygen species (ROS) in fungal cells; hence, we investigated whether *S. cerevisiae* cells produced ROS after treatment with Ppdef1. Cells with FM-464-labelled vacuoles were treated with Ppdef1 in the presence of dihydrorhodamine, (DHR123), a ROS sensitive dye, to monitor ROS production. ROS was detected ~3–7 min after treatment with Ppdef1 (Figure 3C). The small multiple vacuoles that were present in the cells before the addition of Ppdef1 fused into a single large vacuole at 14–16 min (Figure 3B,D). After the fusion of the vacuoles, small puncta of Ppdef1 formed on the cell surface (Figure 3B,E), and SYTOX blue, the cell death stain, entered the cells, showing the plasma membrane had been permeabilized (Figure 3E,F). The vacuole was then completely disrupted, and SYTOX blue was located throughout the dead cell (Figure 3F).

### 3.5. Membrane Permeabilization

**Figure 4:**
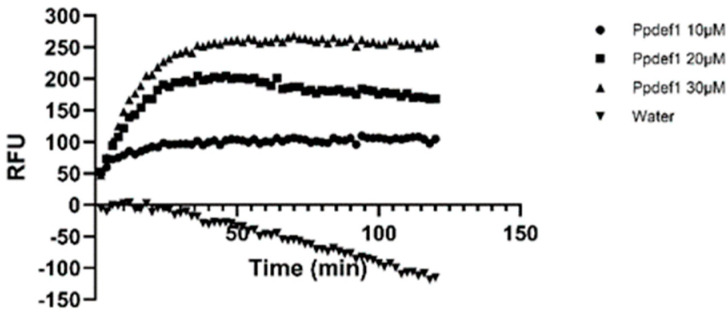
Ppdef1 permeabilizes the membrane of *S. cerevisiae*. Membrane permeabilization by Ppdef1 was monitored kinetically using SYTOX green. *S. cerevisiae* cells treated with 0, 10, 20 or 30 µM Ppdef1 in ½ PDB, and fluorescence was monitored every 2 min over a 120 min period. After treatment with 10 µM Ppdef1, there was an initial increase in fluorescence that plateaued after ~10 min. Yeast cells treated with 20 and 30 µM Ppdef1 showed a rapid increase in fluorescence that reached a maximum after ~40 min for the 30 µM treatment and ~60 min for the 20 µM treatment. The decrease in observed fluorescence in the water treatment was due to the quenching of the background fluorescence over time.

The Ppdef1 permeabilization of the membrane that was observed in microscopy was confirmed using the non-membrane permeable fluorescent dye SYTOX green in a microtitre plate assay. SYTOX green was added to *S. cerevisiae* cells prior to the addition of Ppdef1. Upon the permeabilization of the plasma membrane, SYTOX green enters the cell, binds to nucleic acids and becomes fluorescent. This fluorescence was monitored kinetically by measuring the emission at 535 nm after excitation with 488 nm light. Ppdef1 rapidly permeabilized the membranes of *S. cerevisiae* cells (within 20 min) in a concentration-dependent manner (Figure 4).

### 3.6. ROS Detection

**Figure 5:**
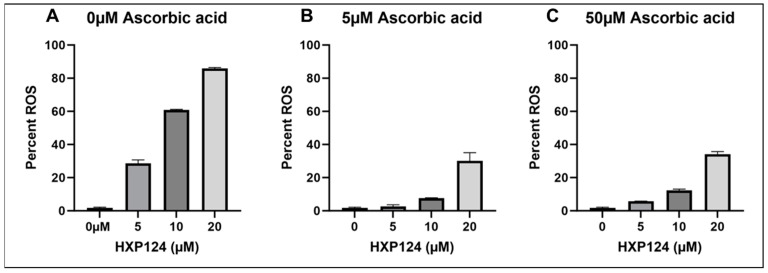
Ppdef1 induces the production of ROS in yeast. Yeast cells were treated with increasing concentrations of Ppdef1 in the presence of the ROS-specific probe, DHR123 and 0 µM (**A**), 5 µM (**B**) or 50 µM (**C**) ascorbic acid. ROS production was assessed with flow cytometry. ROS-positive cells were identified using a cut-off for cellular fluorescence, and the percentage of ROS-positive cells in each treatment was calculated relative to the no Ppdef1 ascorbic acid control. Samples were run in triplicate. The average percentage of ROS positive cells across the three replicates is presented in the bar graph with error bars representing standard deviation.

ROS production in response to Ppdef1 treatment was also confirmed with flow cytometry using the ROS-specific fluorescent probe, DHR123 (Life Technologies, Victoria, Australia). In the presence of ROS, DHR123 is converted to rhodamine 123, which has a fluorescence excitation/emission maximum at 507/529 nm. Ppdef1 induced the production of ROS in yeast cells in a concentration-dependent manner (Figure 5). Treatment with ascorbic acid at 5 µM or 50 µm reduced the amount of ROS produced in *S. cerevisiae* cells, but ROS was still produced at Ppdef1 concentrations of >10 µM (Figure 5B,C), albeit at a reduced level. Excess ascorbic acid (50 µM) had no extra effect on ROS accumulation compared to that at 5 µM concentration.

### 3.7. Screening the Non-Essential S. cerevisiae Deletion Collection

Having established that Ppdef1 enters the cells, a screen of the *S. cerevisiae* haploid non-essential gene deletion collection was performed to identify a potential intracellular target. A fitness profile for Ppdef1 was generated by screening the 5000 strains in the collection in the presence of 2.6 µM Ppdef1. This concentration, chosen because it inhibited 50% of the growth of wild-type *S. cerevisiae*, strain BY4741, was used to treat a pool of the collection in triplicate alongside the wild-type strain. The functional enrichment of strains that were either more resistant or more sensitive to Ppdef1 was performed using FunSpec [37]. The more resistant and more sensitive strains were selected if they had fitness scores of >1.5 or <−1.5, respectively, in relative abundance in the Ppdef1-treated pool compared to the control (*p* value cut-off of 0.05). Strains with increased resistance to Ppdef1 (135 in total) were enriched for deletions in genes that function in mitochondrial respiration, mitochondrial transport, mitochondrial inner membrane and sphingolipid biosynthesis (Table S1). The strains that were more sensitive to Ppdef1 (434 in total) were enriched for genes involved in cell wall biogenesis and organisation, chitin catabolic processes, phosphatase activator activity, polysaccharide metabolism, various endoplasmic reticulum and golgi-related functions and the AP2 and 3 adaptor complexes (Table S2).

### 3.8. Strains with Deletions in Genes with Mitochondrial Function Are Resistant to Ppdef1

**Figure 6:**
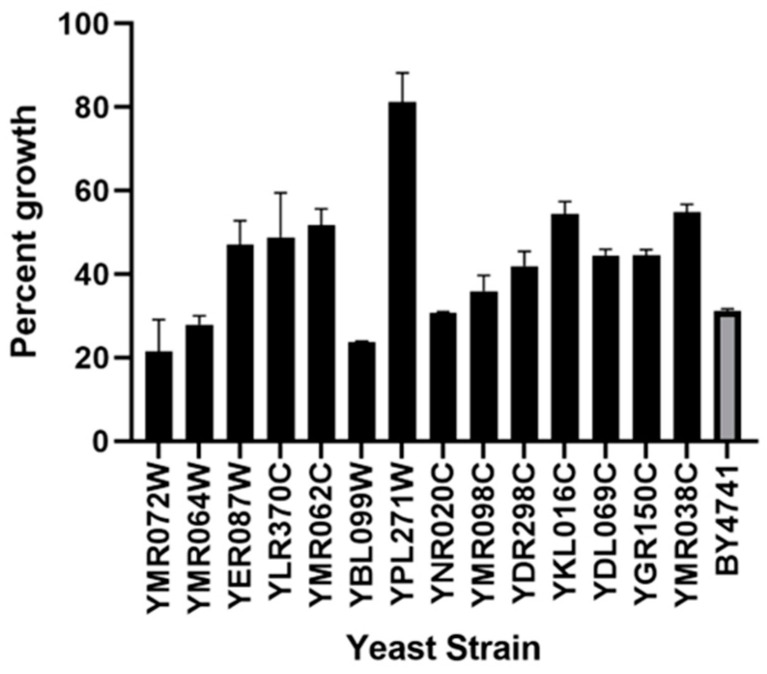
Resistance to Ppdef1 in strains with deletions in genes that function in the mitochondria. Strains with specific mitochondrial gene deletions were grown in the presence and absence of 1 µM Ppdef1 for 16 h. Growth of each strain in the presence of Ppdef1 was normalised to the growth of that strain with no added defensin. Most of the deletion strains grew better than the wild-type strain, BY4741, in the presence of 1 µM Ppdef1, indicating that they are more resistant to the defensin except for *ymr072W*Δ, *ymr064W*Δ and *ybl099W*Δ*,* which were more sensitive to Ppdef1 than the wild type. *Ynr020C*Δ behaved the same as the wild type.

To confirm the results of the high-throughput deletion screen, the 14 yeast strains with deletions in genes that function in the mitochondria were retrieved from the deletion set and assayed for Ppdef1 resistance (Table 2). All these strains, except for *ynr020c*Δ *ymr072W*Δ, *ymr064W*Δ and *ybl099W*Δ*,* had improved growth compared to the wild type in the presence of 1 µM Ppdef1, confirming that they are more resistant to Ppdef1 (Figure 6). The most striking increase in tolerance was observed in mutants associated with F1-F0 ATP synthase.

### 3.9. Ppdef1 Induces the Hyperpolarization of the Mitochondrial Membrane

**Figure 7:**
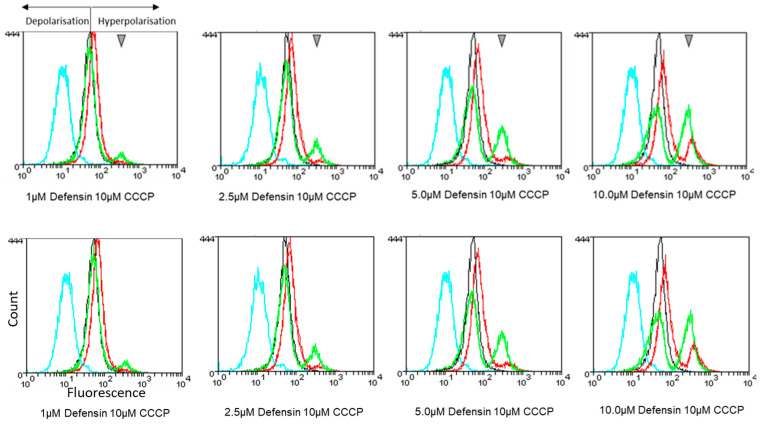
Hyperpolarization of the mitochondrial membrane by Ppdef1. Mitochondrial membrane potential was assessed using tetramethylrhodamine (TMRM), a dye that fluoresces when taken up by mitochondria. The intensity of fluorescence is proportional to the mitochondrial membrane potential. *S. cerevisiae* cells were treated with 1, 2.5, 5.0 or 10 µM Ppdef1 or the plant defensin NaD1. In the graphs above, black lines are untreated cells in the presence of TMRM, blue lines are cells treated with 10 µM CCCP in the presence of TMRM, red lines are cells treated with Ppdef1 defensin in the presence of TMRM and green lines are cells treated with NaD1 defensin in the presence of TMRM. The shift to the left in the CCCP treatment line (blue) with respect to the untreated control line (black) is indicative of mitochondrial membrane depolarization. The shift to the right in the Ppdef1 treatment lines (red) with respect to the untreated control line (black) is indicative of mitochondrial membrane hyperpolarisation. TMRM permeabilized cells are highlighted with grey triangles.

Several strains with increased resistance to Ppdef1 were identified in the screen that had deletions in mitochondrial genes. This prompted us to ask what happens to the mitochondria when cells are exposed to Ppdef1. The effect of Ppdef1 on mitochondrial membrane potential was investigated with flow cytometry using the fluorescent mitochondrial membrane potential indicator tetramethylrhodamine (TMRM). Ppdef1 induced the hyperpolarization of the mitochondrial membrane (Figure 7).

Defensins can have different mechanisms of action in different species of fungi [40,41]; hence, we asked whether the mechanism of action in *S. cerevisiae* was similar as that in *Trichophyton rubrum*. *T. rubrum* spores were too small for the analysis of ROS or permeabilization studies using flow cytometry; therefore, the mechanism of action of Ppdef1 on *T. rubrum* was examined using confocal microscopy on *T. rubrum* hyphae.

### 3.10. Ppdef1 Binds to the Surface and Permeabilizes the Plasma Membrane of Trichophyton rubrum 

**Figure 8:**
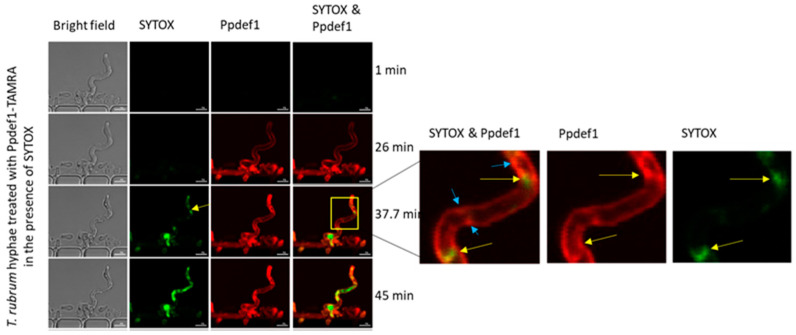
*T. rubrum* cell surface binding and membrane permeabilization by Ppdef1. Ppdef1-TAMRA and SYTOX green were added to *T. rubrum* hyphae, and their location was monitored over time using confocal microscopy. Ppdef1-TAMRA, initially bound to the surface of *T. rubrum* hyphae, formed punctate structures (highlighted in zoomed in box with blue arrows), and SYTOX permeabilized the fungal plasma membrane and are indicated by yellow arrows. Scale bars represent 5 µm.

The location of Ppdef1 in *T. rubrum* hyphae was monitored using Ppdef1 labelled with the red-fluorescent tag 5-Carboxy-tetramethylrhodamine N-succinimidyl ester (TAMRA) (Ppdef1-TAMRA). Membrane permeabilization was monitored simultaneously using the green, fluorescent cell membrane integrity marker, SYTOX green (Figure 8). Ppdef1 bound to the surface of *T. rubrum* after 26 min of treatment. Ppdef1 then accumulated in punctate structures on the edge of the hyphae and intracellularly. After 35 min, SYTOX was detected inside the fungal hyphae, indicating that the plasma membrane had been compromised. As time progressed, both Ppdef1 and SYTOX continued to accumulate in the cytoplasm.

### 3.11. Ppdef1 Induces ROS Production in T. rubrum

**Figure 9:**
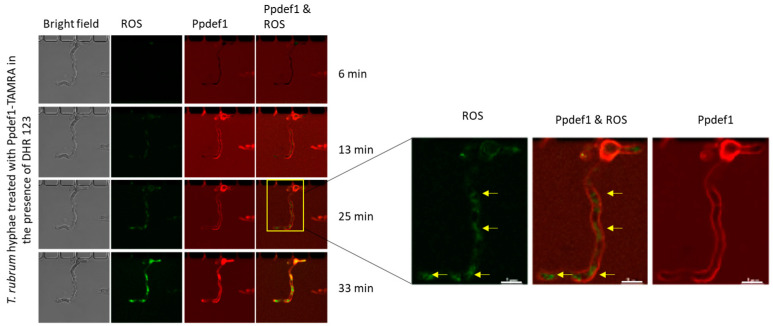
Ppdef1 induces ROS production in *T. rubrum. T. rubrum* hyphae were treated with Ppdef1-TAMRA in the presence of DHR 123. Confocal microscopy was used to monitor the location of Ppdef1-TAMRA (red) and fluorescence of the ROS indicator dye, DHR 123 (green). Ppdef1-TAMRA was bound to the cell surface at 13 min. ROS was detected at 25 min. Yellow arrows highlight the green fluorescence within the hyphae indicating the sites of ROS production. Scale bars represent 5 µm.

ROS production in response to Ppdef1 treatment was also assessed in *T. rubrum.* Since *T. rubrum* hyphae are not amenable to flow cytometry, the production of ROS was monitored using confocal microscopy (Figure 9). The binding of Ppdef1 to the cell surface preceded the production of ROS. The ROS were located in specific regions of the hyphae, but these regions were not identified.

## 4. Discussion

Ppdef1, like many other plant defensins, rapidly kills fungal cells (Figure 3). Ppdef1 was more potent than the four defensins NaD1, ZmD32, NbD6 and NaD2 on *Trichophyton rubrum*, *Trichophyton mentagrophytes*, *Candida auris* and *Cryptococcus neoformans* and has no antibacterial activity (Table 1). Several plant defensins permeabilize the plasma membrane of the fungus [34], leading to the leakage of cellular contents, the disruption of ion gradients and the loss of membrane potential, all of which can lead to cell death. In this study, we report that fungal cells *S. cerevisiae* and *T. rubrum* produce ROS, when they are treated with Ppdef1, and the plasma membrane is compromised (Figure 3, Figure 5, Figure 8 and Figure 9). A screen of the *S. cerevisiae* non-essential gene deletion library identified the involvement of the mitochondria as part of the mechanism of action for Ppdef1 (Table 2). Furthermore, the mitochondrial membrane potential (MMP) is hyperpolarised in yeast cells treated with Ppdef1 (Figure 7).

The sequence of Ppdef1 is very different to other plant defensins, especially loop 5, which is often crucial for lipid binding (Figure 1). The loop region between strands β2 and β3 has been implicated in the antifungal activity and lipid binding of defensins through the ‘cationic grip’ formed by the loop 5 sequences in the dimer [9]. The defensins MtDef4, NaD2, ZmD32 and NbD6 have the loop 5 sequence RGFRRR with a charge of +3.9. Defensins with this loop sequence bind preferentially to PA [11,20] largely due to the charge interaction. Charge interactions with specific lipids are important in membrane disruption [10]. Sagram and coworkers abolished the ability of MtDef4 to enter fungal hyphae when they replaced RGFRRR with AAAARR or RGFRAA, both of which have a reduced charge from +3.9 to +1.9 [20]. NaD1 with the loop sequence SKILRR has a loop 5 charge of +2.9, and its structure in complex with PI(4, 5)P_2_ has been solved, revealing the role of loop 5 in the formation of the cationic grip and lipid binding [9]. Binding to PI(4,5)P_2_ mediates NaD1 oligomerisation and membrane permeabilization [9,23]. In contrast, loop 5 of Ppdef1, QLKGFLNSV, is less charged than these defensins at +0.9, so it is less likely to bind strongly to negatively charged lipids as observed in the analysis with the phospholipids. However, although the SphingoStripTM did not reveal any sphingolipid partners (Figure 2), the interaction with a specific fungal sphingolipid cannot be discounted. The screen against the *S. cerevisiae* deletion library identified strains with increased resistance to Ppdef1 that had deletions of genes involved in sphingolipid biosynthesis (Table 2). Furthermore, interactions between defensins and sphingolipids have been reported previously. The dahlia defensin, DmAMP1, binds to the sphingolipid M(IP)_2_C, increasing plasma membrane permeability, which in turn disrupts essential cellular processes [39]. The charge of the DmAMP1 loop 5 sequence is +2.4. Other than sphingolipids, no other similarities in the mechanisms of action were observed between DmAMP1 and Ppdef1. The radish defensin RsAFP2 directly binds the sphingolipid GlcCer for activity, and like Ppdef1, the charge of the loop 5 sequence NYVFPAHK is low at +1.1. It is thus conceivable that Ppdef1 requires sphingolipids for antifungal activity and should be tested again with fungal sphingolipids not represented on the SphingoStripTM. The activity of Ppdef1 against *S. cerevisiae* which lacks GlcCer indicates that GlcCer is not the target for Ppdef1.

Sphingolipids are important targets for some antifungal molecules. They play important roles in cellular functions and signal transduction pathways and blockage would inhibit growth and induce cell death [42,43]. YGR143W, the strain with the second highest resistant fitness score of all the strains treated with Ppdef1, has a deletion in the gene that encodes a transmembrane glucosidase involved in sphingolipid biosynthesis, cell wall organisation and 1,6-β-glucan biosynthesis. Furthermore, most of the strains with defective genes in sphingolipid biosynthesis, especially those responsible for the biosynthesis of IPCs, MIPCs and M(IP)_2_Cs, were more resistant to Ppdef1. Sphingolipids are thus important for the antifungal activity of Ppdef1, but it is not clear whether they are direct targets or have another function. For example, sphingolipids affect many aspects of endocytosis [44], and defects in endocytosis may affect uptake and hence the toxicity of Ppdef1. This is supported by the observation that the strains with deletions encoding the v-SNARE proteins, YAL030W and YOR327C, had a more resistant fitness score of 1.4 or were more sensitive (−0.14) to Ppdef1, respectively, indicating that endocytosis may internalise Ppdef1. Further studies are required for a better understanding of the role of endocytosis in Ppdef1’s antifungal activity.

The screening of the *Saccharomyces cerevisiae* non-essential deletion library is a useful tool for the identification of cellular components involved in the mechanism of action for plant defensins. Funspec analysis of the strains with fitness scores greater than 1.5 revealed the potential roles of the mitochondria, the respiratory chain, and the mitochondrial proton-transporting ATP synthase complex in Ppdef1 activity. The F1-F0 ATPase (highlighted in grey in Table 2) is a protein complex in the mitochondrial membrane that uses the proton gradient established by the electron transport chain to drive ATP biosynthesis, providing cellular energy. The enhanced resistance to Ppdef1 in strains with gene deletions that affect F1-F0 ATPase genes led to the hypothesis that Ppdef1 may affect the activity of the F1-F0 ATPase and hence mitochondrial membrane potential. Other molecules such as oligomycin and benzodiazepine Bz-423, which inhibit the action of the F1F0 ATPase, also induce mitochondrial membrane hyperpolarization [45]. Mitochondrial membrane hyperpolarization technically refers to the increased relative negative charge in the interior of the mitochondria. Functionally, it is an accumulation of protons in the intermembrane space that results from a lack of proton import into the mitochondria through the ATPase, while the electron transport chain continues to pump protons into the intermembrane space. Ppdef1 enters the cell, causing the hyperpolarisation of the MMP and initiating ROS production in the mitochondria. The MMP is important in regulating ROS levels in the mitochondria and the cell with an exponential dependence of mitochondrial ROS generation, so much so that a small increase in MMP causes a significant increase in ROS production by mitochondria [46]. Two antifungal plant defensins, Vu-Def (*Vigna unguiculata defensin)* [47] and aDef defensin from avocado fruit (*Persea americana* var. drymifolia) [48], and several antifungal molecules have been reported to decrease the mitochondrial membrane potential. Only one plant defensin, RsAFP2, has been reported to induce the hyperpolarisation of the mitochondrial membrane [49] as well as several other antifungal molecules including Plagiochin E [50] and PAF [51]. The antioxidant, ascorbic acid, blocks ROS production using these molecules preventing cell death [29,49]. ROS production was also blocked by ascorbic acid at low concentrations of Ppdef1, but a tenfold increase in ascorbic acid concentration did not reduce ROS production to a greater extent (Figure 5), indicating that there are multiple pathways by which ROS is produced in response to Ppdef1, at least one of which is not affected by ascorbic acid. This is further supported by the observation that ascorbate was less effective at reducing ROS production at higher concentrations of Ppdef1 (Figure 5). A similar phenomenon has been reported for miconazole. Miconazole treatment of sessile *C. albicans* in the presence of ascorbic acid resulted in a reduction of ROS production. However, cell death still occurred, suggesting a second mechanism for its antifungal activity that is not counteracted by ascorbic acid [52].

Several of the gene deletions associated with mitochondrial function and increased resistance to Ppdef1 function in programmed cell death (PCD) have been identified in screens with other defensins [16,53,54]. Thus, Ppdef1 is likely to have an upstream target/s that initiates PCD in yeast. The removal of strains specific to PCD revealed other deletion strains with improved resistance to Ppdef1 that were unique to Ppdef1. These included MDM34, MDM10, MDM12, MMM2, AIM3, LSB5, SWF1, RVS167 and AIM21 genes involved in the altered inheritance rate of mitochondria and CDC26, SSE1, SWM1 and MND2 genes involved in cell cycle division. 

A blastp search of the non-redundant protein database with the loop 5 sequence, QLKGFLNSV, of Ppdef1 revealed 100% sequence identity to several enzymes. These enzymes included helicases, glycosyltransferases, peroxidase, oxidoreductase, lipase, ligases and metallohydrolase. The nine amino acid sequence of loop 5 could be required for binding to substrates, and perhaps, the loop 5 is binding to the active site of a specific enzyme or substrate in sphingolipid biosynthesis. The valine tRNA ligase from several bacterial species also had 100% sequence identity to the loop 5 sequence. Furthermore, a sequence alignment of the more resistant strain to Ppdef1 with a gene deletion in the mitochondrial phenylalanine tRNA ligase activity with the loop 5 sequence identified a motif, GFLXSX. 

Ppdef1 bound with high affinity to chitin and yeast β-glucan and the cell surface. Confocal microscopy with Ppdef1-FAM/TAMRA-treated yeast cells revealed binding and accumulation on the cell surface (Figure 3). The cell wall of fungi is composed predominantly of chitin and 1,6- and 1,3-β-glucan and protects the fungal cell from cationic peptides in the presence of salt [31,55]. The outer cell wall surface also has a high proportion of mannoprotein [56]. Although in vitro mannoprotein binding was not tested, it is conceivable that Ppdef1 binds to mannoprotein due to the accumulation of Ppdef1 at the cell surface. A sequence alignment of Ppdef1 with the chitin-binding domain CBD sequence from several chitin-binding proteins did not detect the domain [57]. NaD1 also binds to polysaccharides and lacks a CBD. However, the removal of the cell wall improved the activity of NaD1 [31]. Ppdef1 could be concentrated on the cell wall of the fungal hyphae before it interacts with the plasma membrane. It would be interesting to see if removing the cell wall prevents Ppdef1 activity, or like NaD1, it improves the antifungal activity. 

Similar mechanisms of action were observed when *S. cerevisiae* cells or *T. rubrum* hyphae were treated with Ppdef1. ROS was produced and permeabilization occurred in both (Figure 9). Mitochondrial membrane potential and ascorbic acid recovery were too difficult to measure in *T. rubrum* as hyphae could not be subjected to FACS analysis. Due to the small size of the hyphae, confocal microscopy did not provide conclusive results for MMP hyperpolarisation or recovery with ascorbic acid. 

**Figure 10:**
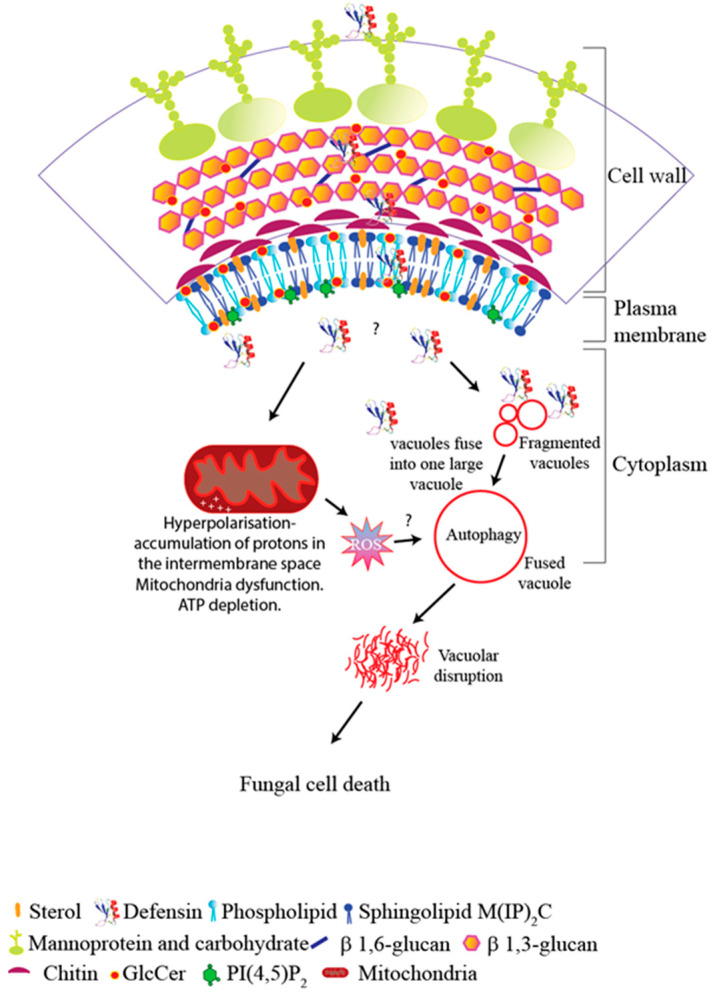
Model of the proposed mechanisms of action of Ppdef1. Ppdef1 binds to the fungal cell wall and is endocytosed from the plasma membrane. It penetrates the cytoplasm and targets mitochondria, causing the hyperpolarisation of the mitochondrial membrane potential and ROS, induces programmed cell death, induces vacuolar fusion and causes fungal cell death. This model is an addition to the previously described mechanisms of action of defensins [8].

A model of the proposed mechanism of Ppdef1 is presented in Figure 10. Ppdef1 binds to the cell surfaces of *S. cerevisiae cells* and *T. rubrum* likely via mannoprotein, chitin and β-glucan in the cell wall. It then penetrates the plasma membrane via endocytosis as reported for NaD1 [57] and enters the cytoplasm. Upon intracellular entry, Ppdef1 activates programmed cell death potentially via mitochondrial dysfunction or the impairment of cell cycle division, where it causes the hyperpolarisation of the MMP and ROS production. This in turn triggers vacuolar fusion, membrane permeability and cell death (Figure 10).

## Figures and Tables

**Table 1:** IC50 µg/mL of five plant defensins against several pathogenic fungi and pathogenic bacteria.

Pathogen	Ppdef1 IC50 (µg/mL)	NaD1 IC50 (µg/mL)	ZmD32 IC50 (µg/mL)	NbD6 IC50 (µg/mL)	NaD2 IC50 (µg/mL)
*Trichophyton rubrum*	4.6	7.9	-	22.5	>64.0
*Trichophyton mentagrophytes*	8.4	6.5	-	13.4	25.8
*Candida albicans*	3.4	12.2	3.9	1.6	-
*Candida tropicalis*	1.9	2.7	0.7	2.1	5.8
*Candida glabrata*	15.4	13.8	6.5	1.9	23.2
*Candida auris*	1.3	-	3.6	2.4	-
*Candida krusei*	3.0	-	3.5	2.0	-
*Cryptococcus neoformans*	2.7	8.8	-	7.1	4.2
*Cryptococcus gattii*	38.9	9.0	-	19.5	20.0
*Aspergillus niger*	42.7	11.1	-	44.5	50.0
*Microsporum fulvum*	7.1	22.3	-	11.9	37.9
*Saccharomyces cerevisiae*	8.8	19.0	19.7	19.7	-
*Fusarium graminearum*	24.7	2.7	5.5	-	10.5
*Fusarium oxysporum*	7.7	-	-	19.5	26.3
*Escherichia coli*	>200	38.0	5.5	-	-
*Staphylococcus aureus*	>200	26.5	8.2	-	-
*Pseudomonas aeruginosa*	>200	22.8	9.3	-	-
*Bacillus subtilis*	>200	10.6	2.2	-	-

**Table 2:** Strains with increased resistance to Ppdef1 and those selected for the validation of Ppdef1 resistance. The fitness score is calculated as the base 2 logarithm of the average ratio of the normalized number of barcode counts in the Ppdef1-treated pool compared to the control pool. Grey shading indicates strains related to the F1-F0 ATP synthase. Fitness score indicates fold-change relative to wild type. Strains tested for validation are highlighted with ‘*’.

Systematic Name	Gene	Fitness Score	Gene Description
YGR143W	SKN1	3.82	Protein involved in sphingolipid biosynthesis
YDR298C *	ATP5	3.38	Subunit 5 of the stator stalk of mitochondrial F1F0 ATP synthase
YMR098C *	ATP25	3.22	Mitochondrial protein required for the stability of Oli1p
YDR072C	IPT1	3.00	Inositolphosphotransferase; involved in synthesis of mannose-(inositol-P)2-ceramide (M(IP)2C), the most abundant sphingolipid
YPL057C	SUR1	2.45	Mannosylinositol phosphorylceramide (MIPC) synthase catalytic subunit
YMR072W *	ABF2	2.22	Mitochondrial DNA-binding protein
YKL016C *	ATP7	2.08	Subunit d of the stator stalk of mitochondrial F1F0 ATP synthase
YPL271W *	ATP15	1.82	Epsilon subunit of the F1 sector of mitochondrial F1F0 ATP synthase
YNR020C *	ATP23	1.81	Putative metalloprotease of the mitochondrial inner membrane
YER087W *	AIM10	1.60	Protein with similarity to tRNA synthetases
YGR150C *	CCM1	1.43	Mitochondrial 15S rRNA-binding protein
YMR038C *	CCS1	1.40	Copper chaperone for superoxide dismutase Sod1p; involved in oxidative stress protection; located in mitochondrial inner membrane
YMR062C *	ARG7	1.39	Mitochondrial ornithine acetyltransferase
YBL099W *	ATP1	1.26	Alpha subunit of the F1 sector of mitochondrial F1F0 ATP synthase
YLR370C *	ARC18	1.24	Subunit of the ARP2/3 complex
YMR064W *	AEP1	1.23	Protein required for expression of the mitochondrial OLI1 gene which encodes subunit 9 of F1-F0 ATP synthase
YDL069C *	CBS1	1.17	Mitochondrial translational activator of the COB mRNA

## Data Availability

Data are available on request due to privacy restrictions. The data presented in this study are available on request from the corresponding author. The data are not publicly available due to company policy.

## Supplementary Materials

The following supporting information can be downloaded at: https://www.mdpi.com/article/10.3390/jof10010054/s1, Table S1: Functional enrichment of strains with increased resistance to Ppdef1 identified with Funspec title. Table S2 Functional enrichment of strains with decreased resistance to Ppdef1 identified with Funspec.
