# Supplementary material for: Hyperpolarisation of Mitochondrial Membranes Is a Critical Component of the Antifungal Mechanism of the Plant Defensin, Ppdef1
Source: J Fungi (Basel). 2024 Jan 7;10(1):54. doi: 10.3390/jof10010054 (PMC10817573; doi:10.3390/jof10010054)
Supplement: Supplementary file 1 [file jof-10-00054-s001.zip › jof-2766550-supplementary.pdf]

Table S1 . Functional enrichment of strains with increased resistance to ppdef1 identified with Funspec

The fitness score for strains with resistance to Ppdef1 > 1.5 was analysed. Number of Genes in each Database: 6603. **k**: number of genes from the input cluster in given category. **f**: number of genes total in given category. The p-values represent the probability that the intersection of a given list with any given functional category occurs by chance.

Category	P value	In category from cluster	k	f
**GO molecular function**				
hydrogen ion transporting ATP synthase activity, rotational mechanism [GO:0046933]	0.004	ATP5 ATP7 ATP15	3	17
kinase activity [GO:0016301]	0.006	VPS15 CHK1 FAB1 HXK2 PFK1 PTK2 VPS34 SKY1 PHO85 THI6	10	206
hydrogen ion transporting ATP synthase activity, rotational mechanism [GO:0046933]	0.004	ATP5 ATP7 ATP15	3	17
**GO biological Process**				
negative regulation of phosphate metabolic process [GO:0045936]	0.0004	PHO80 PHO85	2	2
negative regulation of calcium-mediated signaling [GO:0050849]	0.0004	PHO80 PHO85	2	2
regulation of establishment or maintenance of cell polarity [GO:0032878]	0.002	PCL2 PHO85	2	4
negative regulation of macroautophagy [GO:0016242]	0.002	PHO80 PHO85	2	4
sphingolipid biosynthetic process [GO:0030148]	0.002	IPT1 SKN1 SUR1	3	15
protein refolding [GO:0042026]	0.003	MDJ1 CPR7 SSE1	3	16
polyamine transport [GO:0015846]	0.003	AGP2 PTK2	2	5
ATP synthesis coupled proton transport [GO:0015986]	0.004	ATP5 ATP7 ATP15	3	17
negative regulation of catalytic activity [GO:0043086]	0.005	INH1 PRB1	2	6
phosphorylation [GO:0016310]	0.006	VPS15 CHK1 FAB1 HXK2 PFK1 PTK2 VPS34 SKY1 PHO85 THI6	10	206
phosphatidylinositol phosphorylation [GO:0046854]	0.009	FAB1 VPS34	2	8
regulation of establishment or maintenance of cell polarity [GO:0032878]	0.002	PCL2 PHO85	2	4
**GO cellular components**				
mitochondrial inner boundary membrane [GO:0097002]	0.001	YTA12 MGM1	2	3

mitochondrial proton-transporting ATP synthase, stator stalk [GO:0000274]	0.001	ATP5 ATP7	2	3
mitochondrial inner membrane [GO:0005743]	0.002	COQ1 MSS2 ATP5 FLX1 ATP7 YTA12 ATP25 COX5A ATP23 MGM1 ATP15	11	204
phosphatidylinositol 3-kinase complex II [GO:0034272]	0.002	VPS15 VPS34	2	4
phosphatidylinositol 3-kinase complex I [GO:0034271]	0.002	VPS15 VPS34	2	4
clathrin-coated vesicle [GO:0030136]	0.003	CHS6 TVP18 LDB19	3	16
cyclin-dependent protein kinase holoenzyme complex [GO:0000307]	0.004	PCL2 PHO80 PHO85	3	17
mitochondrial proton-transporting ATP synthase complex [GO:0005753]	0.005	ATP5 ATP7	2	6
proton-transporting ATP synthase complex, catalytic core F(1) [GO:0045261]	0.005	ATP5 ATP15	2	6
extrinsic to mitochondrial inner membrane [GO:0031314]	0.005	MSS2 MDJ1 MGM1	3	19
mitochondrial inner boundary membrane [GO:0097002]	0.001	YTA12 MGM1	2	3
**MIPS functional classification**				
energy generation (e.g. ATP synthase) [02.45.15]	0.0006	INH1 ATP5 ATP7 ATP15	4	21
vitamine/cofactor transport [20.01.25]	0.002	AGP2 FEN2 FLX1	3	14
mitochondrial transport [20.09.04]	0.004	MSS2 ATP5 SMF2 FLX1 ATP7 YTA12 ATP15	7	104
electron transport and membrane-associated energy conservation [02.11]	0.005	INH1 ATP5 ATP7 COX5A ATP15	5	58
electron transport [20.01.15]	0.005	INH1 ATP5 ATP7 COX5A GRX5 ATP15	6	83
respiration [02.13]	0.005	ATP5 ATP7 IRC21 YTA12 ATP15	5	59
transcription activation [11.02.03.04.01]	0.008	THI2 THI3 HAP2 MGA2	4	42
modification by phosphorylation, dephosphorylation, autophosphorylation [14.07.03]	0.009	VPS15 SLI15 CHK1 REG1 PTK2 SAP190 VPS34 SKY1 PHO85	9	186
phospholipid metabolism [01.06.02.01]	0.010	COQ1 CST26 IPT1 ISC1 SUR1	5	68

Table S2 Functional enrichment of strains with increased sensitivity to ppdef1 identified with Funspec

The fitness score for strains with resistance to Ppdef1 <1.5 was analysed. Number of Genes in each Database: 6603.

k: number of genes from the input cluster in given category. **f**: number of genes total in given category. The p-

values represent the probability that the intersection of a given list with any given functional category occurs by chance.

Category	P value	In category from cluster	k	f
**GO molecular function**				
phosphatase activator activity [GO:0019211]	0.001	TOF2 WHI2 RRD2	3	4
siderophore transmembrane transporter activity [GO:0015343]	0.004	ARN1 ARN2	2	2
mannan endo-1,6-alpha-mannosidase activity [GO:0008496]	0.004	DCW1 DFG5	2	2
chitin deacetylase activity [GO:0004099]	0.004	CDA1 CDA2	2	2
transferase activity, transferring glycosyl groups [GO:0016757]	0.004	PMT2 PMT1 KRE2 DIE2 YUR1 PNP1 APT1 ALG9 ALG6 ALG8 ALG5 GPH1	12	80
**GO biological process**				
fungal-type cell wall biogenesis [GO:0009272]	2.155e-05	FLC2 ROT2 RIM101 DCW1 DFG5 RIM21	6	11
ubiquitin-dependent protein catabolic process via the multivesicular body sorting pathway [GO:0043162]	0.0002	STP22 VPS25 SNF7 VPS36 VPS20 SNF8	6	15
cellular cell wall organization [GO:0007047]	0.0004	ECM33 SBE2 SMI1 ECM34 SBE22 CHS7 DCW1 ECM7 GAS3 DFG5 ZDS1 SCW10 GAS1 KRE1 WSC3	15	89
chromatin silencing [GO:0006342]	0.0004	SPT4 SDS3 CAC2 ZDS1 GAS1 HST3	6	17
negative regulation of transcription from RNA polymerase II promoter by glucose [GO:0000433]	0.0005	MIG2 VPS25 VPS36 SNF8	4	7
chitin catabolic process [GO:0006032]	0.001	CTS2 CDA1 CDA2	3	4
polysaccharide catabolic process [GO:0000272]	0.002	CTS2 CDA1 CDA2	3	5
protein folding in endoplasmic reticulum [GO:0034975]	0.004	EMC1 EMC4 AIM27 SCJ1	4	11
AMP biosynthetic process [GO:0006167]	0.004	ADO1 APT1	2	2
ER-associated misfolded protein catabolic process [GO:0071712]	0.004	PMT2 PMT1	2	2
carbohydrate catabolic process [GO:0016052]	0.004	DCW1 DFG5	2	2
budding cell bud growth [GO:0007117]	0.004	REI1 BEM2 DCW1 ELM1 DFG5	5	18
sporulation resulting in formation of a cellular spore [GO:0030435]	0.005	RRT12 TRS85 CTS2 RMD6 IME4 RSC1 SPO11 SSP1 SPO22 CDA1 CDA2 VAN1 SSP2 RIM20	14	103
protein processing [GO:0016485]	0.005	RIM8 RIM13 DFG16 RIM20	4	12
carbohydrate metabolic process [GO:0005975]	0.006	ROT2 CTS2 MIG2 PCL7 IMA5 CDA1 CDA2 YLR446W GAS3 SCW10 GAS1 GAL4 GPH1	13	94

ascospore formation [GO:0030437]	0.009	IML3 RIM101 SET2 RIM9 IML3 RIM101 SET2 RIM9 RIM11 YNL194C RIM21 MCK1 SSP2	9	57
**GO cellular component**				
endoplasmic reticulum [GO:0005783]	5.376e-06	PMT2 FRT2 ERV46 FLC2 ROT2 BSD2 EMC1 CWH43 PMT1 TRS85 MSC2 YEA4 YEL043W ERG4 GET1 GUP1 EMC4 NNF2 DIE2 CHS7 APQ12 ICE2 SCP160 PHO86 SOP4 STE24 ILM1 AIM27 GMH1 ERG3 ARV1 SUR4 ERV25 SPC2 VAN1 ERG5 ERG2 SCJ1 SCW10 YNL194C ALG9 MID1 COS10 INP54 ALG6 ALG8 SEY1 YDC1 ALG5 YPR114W	50	416
ESCRT II complex [GO:0000814]	0.0003	VPS25 VPS36 SNF8	3	3
Golgi apparatus [GO:0005794]	0.0004	ERV46 PEP1 APM3 TRS85 SBE2 KRE2 GET1 PSD2 APL6 YHL017W GOS1 SBE22 GVP36 APS3 MTC1 YUR1 ATG27 YKL063C TPO5 BCH2 GMH1 ARV1 ERV25 VAN1 NPR1 GNT1 YDC1	27	213
membrane [GO:0016020]	0.0007	PMT2 FRT2 ERV46 FLC2 SEO1 YAR029W PEP1 FUR4 ECM33 AAC3 MTC4 APM3 BSD2 LDB16 STP22 EMC1 CWH43 CDC50 PMT1 SED1 MSC2 PEX5 MFA1 KRE2 YEA4 VAC8 AFG3 ERG4 GET1 GUP1 HUR1 EMC4 YGR026W NNF2 GTR2 DIE2 APL6 DUR3 YHL017W APM2 GOS1 ARN1 ECM34 YHL044W ARN2 ANS1 CHS7 MTC6 YAP1801 SSP1 TIR3 APQ12 GVP36 ICE2 QDR2 AXL2 COA1 APS3 SCP160 PHO86 TRK1 YUR1 VPS35 YJL163C ATG27 SOP4 APL1 APS2 OPI3 VPS25 STE24 ILM1 VPS70 DCW1 TPO5 STE3 AIM27 BCH2 GMH1 RHO4 PTR2 SNF7 YLR046C ERG3 CSF1 ARV1 SUR4 VPS36 ECM7 ERV25 SPC2 TCB3 ALO1 VAN1 YMR034C RIM9 VPS20 DLT1 YIM1 YMR155W INP2 EAR1 ERG2 GAS3 DFG5 GAS1	134	1671

		AQR1 YNL194C ALG9 MID1 RIM21 KRE1 PDR18 COS10 APM4 INP54 WSC3 MCH4 ALG6 TIR2 DFG16 ALG8 VAM10 VPS17 SEY1 YOR246C TIM18 GNT1 SNF8 NCR1 YDC1 ALG5 YPR114W YPR174C		
endoplasmic reticulum membrane [GO:0005789]	0.0008	PMT2 FRT2 ERV46 FLC2 EMC1 CWH43 PMT1 MSC2 YEA4 GET1 EMC4 NNF2 DIE2 CHS7 ICE2 SCP160 SOP4 STE24 ILM1 AIM27 GMH1 ERG3 ARV1 ERV25 SPC2 VAN1 ERG2 ALG9 INP54 ALG6 ALG8 SEY1 YDC1 ALG5 YPR114W	35	318
AP-2 adaptor complex [GO:0030122]	0.001	APL1 APS2 APM4	3	4
AP-3 adaptor complex [GO:0030123]	0.001	APM3 APL6 APS3	3	4
cytoplasmic mRNA processing body [GO:0000932]	0.001	CCR4 DHH1 SBP1 RPB4 POP2 VTS1 SYH1	7	28
retromer complex [GO:0030904]	0.002	VPS29 VPS35 VPS17	3	5
coated pit [GO:0005905]	0.002	APM2 APL1 APS2 APM4	4	10
intracellular [GO:0005622]	0.003	RPS8A REI1 BRE1 RPP1A TOM1 RML2 RPS26B BEM2 HUL5 MIG2 UGA1 RBG2 RIM101 OCA5 RTT107 RPS4B RPL34B RPS24B HIS5 RPL40A RPS21B APN1 RHO4 PNP1 VPS36 MSS1 MIH1 RPL13B RIM13 RPL9B RPL42A SQS1 IRA2 RPS19A RPS7A REV1 RPL21B USV1	38	381
endosome [GO:0005768]	0.003	STP22 CDC50 AGE1 PSD2 ARN1 ARN2 VPS29 VPS35 VPS25 SNF7 VPS36 VPS20 EAR1 VPS17 SNF8	15	108
chitosan layer of spore wall [GO:0005631]	0.004	CDA1 CDA2	2	2
dolichyl-phosphate-mannose-protein mannosyltransferase complex [GO:0031502]	0.004	PMT2 PMT1	2	2
ER membrane protein complex [GO:0072546]	0.004	EMC1 EMC4 AIM27	3	6
anchored to membrane [GO:0031225]	0.004	ECM33 SED1 ANS1 TIR3 DCW1 GAS3 DFG5 GAS1 KRE1 TIR2	10	61
N-directed glycosylation, deglycosylation [14.07.02.02]	0.0003	KRE2 DIE2 YUR1 VAN1 SCJ1 ALG9 ALG6 ALG8 GNT1 ALG5	10	43

development of asco- basidio- or zygospore [43.01.03.09]	0.0007	ECM33 TRS85 CTS2 IME4 RIM101 SSP1 SPO22 SDS3 SNF7 CDA1 CDA2 VAN1 RIM9 RIM11 RIM13 YNL194C RIM21 MCK1 POP2 SIN3 SSP2 RIM20	22	166
polysaccharide metabolism [01.05.03]	0.001	SMI1 CDA1 CDA2 MSS11 GAS3 SCW10 GAS1 GPH1	8	35
cell wall [42.01]	0.002	ECM33 ROT2 CWH43 SED1 SBE2 CTS2 KRE2 YEA4 BEM2 SMI1 ECM34 SBE22 CHS7 YUR1 HAL5 DCW1 ECM7 VAN1 GAS3 DFG5 GAS1 ALG9 KRE1 WSC3 DFG16	25	213
transcriptional control [11.02.03.04]	0.006	CCR4 ROX3 SIF2 CDC50 PHO2 INO2 HPR1 ARO80 YHP1 UBP3 IME4 MIG2 SPT4 NDT80 RTT107 EGD2 DOT5 SDS3 TPK1 SET2 YJL206C SPT8 HOG1 TOS4 DAT1 SOK2 RIM11 MSS11 SPT21 FAP1 GIS2 CAF120 CSE2 POP2 SIN3 INO4 HST3 ISW2 HHO1 HDA3	40	426
budding, cell polarity and filament formation [43.01.03.05]	0.007	RVS161 CDC50 SBE2 BUD16 BEM2 BUD27 RIM8 KEL2 GIC1 SBE22 YAP1801 AXL2 BNR1 TPK1 ACF4 ELM1 RHO4 SUR4 SOK2 RIM9 MSS11 DFG5 ZDS1 RIM21 DFG16 CKB2 MSB1 BFR1 RIM20 CLN2 SRO7	31	312
